# Sperm Impairment by Sperm Agglutinating Factor Isolated from *Escherichia coli*: Receptor Specific Interactions

**DOI:** 10.1155/2013/548497

**Published:** 2013-07-17

**Authors:** Kiranjeet Kaur, Vijay Prabha

**Affiliations:** Department of Microbiology, Panjab University, Chandigarh 160014, India

## Abstract

In an earlier work done in our laboratory, we have been able to isolate a sperm agglutinating strain of *Escherichia coli* from the semen sample of a male attending infertility clinic. Further, factor responsible for sperm agglutination (SAF) was isolated and purified, and, using SAF as a tool, corresponding SAF binding receptor from human spermatozoa has been purified. Characterization of SAF and SAF binding receptor using MALDI-TOF showed homology to glutamate decarboxylase and MHC class I molecule, respectively. Coincubation of SAF with spermatozoa not only resulted in spermagglutination but could also compromise other sperm parameters, namely, Mg^2+^ dependent ATPase activity and apoptosis. Intravaginal administration of SAF could lead to infertility in Balb/c mice. SAF induced impairment of sperm parameters, and infertility was observed to be due to interaction of SAF with sperm surface receptor component as, when purified receptor was introduced, receptor completely inhibited all the detrimental effects induced by SAF. From these results, it could be concluded that interaction of SAF with spermatozoa is receptor mediated.

## 1. Introduction 

The cause-effect relationship between bacterial infections and infertility is still being debated [1]. Various microorganisms are well-known causative agents of genitourinary infections such as *Escherichia coli*,* Staphylococcus aureus* [2],* Ureaplasma urealyticum*,* Mycoplasma hominis, *and *Chlamydia trachomatis* [3] which have the ability to interact directly with spermatozoa. 

*Escherichia coli *represents the most frequently isolated microorganism in patients with genitourinary infections and male accessory gland infections (MAGIs) [4]. *E. coli* rapidly adheres to human spermatozoa *in vitro*, resulting in agglutination of spermatozoa. A profound decline in motility of spermatozoa is evident over time caused by severe alterations in sperm morphology [5]. Morphological alterations involved all superficial structures of sperm, in particular the plasma membrane of mid piece and neck as well as inner and outer acrosomal membranes of the acrosome indicating that morphological defects might be accounting for the immobilization of spermatozoa by *E. coli*. Thus, it was suggested that *E. coli*/spermatozoa interaction may be a two-step process, that is, adhesion to and subsequent destruction of the sperm membrane [5] pointing towards the presence of receptor-ligand interaction between spermatozoa and *E. coli *playing most imperative role behind the above-mentioned phenomena.

Because the surface of spermatozoa is rich in glycoproteins, even asymptomatic colonization of the male or female genitalia with Enterobacteriaceae may result in similar interactions. Monga and Roberts [6] first provided evidence for bacteria-spermatozoa interactions at the receptor-ligand level. While studying the mechanism of sperm agglutination, they identified two *E. coli* surface adhesions, P-fimbriae and type 1 fimbriae that interact with specific receptors (*α*-galp-1,4-*β*-galp-O-methyl[gal-gal], mannose) leading to agglutination of spermatozoa. Although a number of studies have evaluated the ability of *E. coli* to affect sperm motility by adherence, agglutination, and dialyzable factors, very few have identified the mechanism of interaction between spermatozoa and bacteria.

 Earlier studies in our laboratory have reported the isolation and purification of sperm agglutinating factor (SAF) from *E. coli* [7]. SAF could induce sperm agglutination, death, and morphological changes in human spermatozoa *in vitro*. Fluorescent microscopy revealed the binding of FITC tagged SAF on both sperm head and tail. Further, using SAF, corresponding sperm receptor from spermatozoa has also been extracted and purified [8]. This receptor was capable of blocking sperm agglutination, death, and morphological alterations induced by SAF. In the present study, we investigated the effect of SAF on other sperm parameters, namely, Mg^2+^ dependent ATPase activity and sperm apoptosis *in vitro* along with effect of SAF on fertility outcome in female Balb/c mice *in vivo*. Further, to highlight and understand receptor mediated damage by SAF, blocking studies using principle of competitive inhibition were carried out both *in vitro* and *in vivo.*

## 2. Material and Methods

### 2.1. Animals

Sexually mature 5-6-week old male (25 ± 2 g) and 4-5-week old female (22 ± 2 g) Balb/c mice were used in the present study. The animals were maintained under standard laboratory conditions (12 h light: 12 h darkness cycle) and were housed in polypropylene cages. Food and water were available ad libitum. All the experiments were approved by Institutional Animal Ethical Committee, Panjab University, see no. IAEC/156 dated 25.08.11, and were performed in accordance with the guidelines of the Committee for the Purpose of Control and Supervision of Experiments on Animals (CPCSEA).

### 2.2. Semen Sample

Semen samples were obtained by masturbation, into a sterile wide mouth container, from men undergoing fertility evaluation at Post Graduate Institute of Medical Education and Research (PGIMER), Sector 12, Chandigarh, India. Ejaculates showing normal semen parameters according to World Health Organization [9] criteria were used. The protocols for the present study were approved by Panjab University Institutional Ethics Committee; see letter no. 02/PUIEC dated 25.03.09.

### 2.3. Isolation and Purification of Sperm Agglutinating Factor (SAF) from *E. coli* and Sperm Receptor from Human Spermatozoa

The SAF was extracted and purified from 48 h old cell culture of *E. coli* by the method earlier standardized in the laboratory [7]. Briefly, the cell culture was washed with Phosphate buffered saline (PBS; pH 7.2, 50 mM) and pellet was given salt treatment with 3 M NaCl for 12 h which released the factor into the solution. After dialysis and concentration of salt treated sample, further purification of SAF was carried out using gel permeation chromatography and ion exchange chromatography. 

For isolating SAF binding receptor from human spermatozoa, spermatozoa were washed with PBS and subjected to salt treatment and it was observed that the receptor could be extracted efficiently by 18 h treatment with 2 M NaCl. Purification of SAF binding receptor was done by gel filtration through Sephadex G-200 column and the bioactive fractions (showing blockage of agglutination) were pooled and concentrated against Polyethylene glycol (PEG) at 4°C [8]. 

### 2.4. Chemicals and Kit

All the chemicals and reagents used were of analytical grade and were purchased from Hi-Media and Qualigens Laboratories, India. For Apoptosis, Annexin V-PI staining kit was procured from Sigma Aldrich Chemicals (St. Louis, MO, USA). 

### 2.5. Characterization by Matrix Assisted Laser Desorption Ionisation-Time of Flight (MALDI-TOF) of SAF and Sperm Receptor

The services for MALDI-TOF were outsourced to The Centre for Genomic Applications (TCGA), New Delhi, India for both SAF and SAF binding receptor. For this, the purified proteins were run on a 10% Polyacrylamide gel separately and stained with Coomassie Brilliant Blue. Entire gel was rinsed in ultrapure water for 1-2 h, gel piece containing protein band was excised to 1–1.5 mm cubes and transferred to 1.5 mL microfuge tubes. Gel pieces were washed once with 100 *μ*L HPLC grade water for 10 min followed by destaining using 50% 100 mM ammonium bicarbonate in 50% acetonitrile (ACN) at 37°C for 30 min. Dehydration was done using ACN followed by reduction and alkylation using 10 mM of dithiothreitol/100 mM of ammonium bicarbonate and 50 mM of iodoacetamide/100 mM of ammonium bicarbonate, respectively. The gel piece was washed again with destaining solution followed by dehydration. Gel pieces were rehydrated in ~5 *μ*L trypsin solution (20 ng/*μ*L) and incubated at 37°C for 16 h. Peptide extraction solution (1% trifluoroacetic acid) was added to extract the peptides which were used for subsequent MS analysis. The resulting spectrum was searched for matching proteins in the NCBI database using the MASCOT search engine (Matrix Science). 

### 2.6. Receptor Mediated Sperm Impairment by SAF

#### 2.6.1. Mg^2+^ Dependent Adenosine Triphosphatase (ATPase) Activity

Motile spermatozoa selected by swim-up procedure were washed twice (500 g, 5 min) in phosphate buffered saline (PBS, 50 mM, pH 7.2), and the final working concentration of sperm obtained was adjusted to 1 × 10^8^ spermatozoa/mL in Tris-HCl buffer (0.2 M, pH 7.6). Sonication of washed spermatozoa (1 × 10^8^/mL) was carried out at 50 Hz for 10 min.

To determine effects of SAF on Mg^2+^ dependent ATPase activity, different concentrations of SAF (5, 10, 20, 40, and 80 *μ*g) were added to 0.2 mL of sonicated sperm suspension along with the reaction mixture (0.2 mL Tris-HCl buffer, 0.2 mL of MgCl_2_ (5 mM) and 0.2 mL of ATP (6 mg/mL)) [10, 11]. After incubation at 37°C for 1 h, the reaction was stopped by adding 1 mL of cold 10% trichloroacetic acid (TCA) and then incubated at 4°C overnight for protein precipitation. Also, the evaluation of receptor mediated blockage of Mg^2+^ dependent ATPase activity of spermatozoa was done, for these different concentrations of receptor (50, 75, and 100 *μ*g) were added in combination with SAF (80 *μ*g) and preceded in the same manner as mentioned above. The control tubes contained all the components of the reaction mixture, but TCA was added in the beginning to stop the ATPase activity. Inorganic phosphorus (Pi) released was determined according to the method of Boyce et al. [11]. One unit of ATPase was expressed as the *μ*moles of Pi released after 1 h of incubation at 37°C.

#### 2.6.2. Apoptosis

To study whether SAF induced death was associated with apoptosis and receptor mediated, human semen samples were washed twice in Biggers-Whitten-Whittingham (BWW) medium (pH 7.4). After washing, spermatozoa were divided in aliquots having 4 × 10^6^/mL motile spermatozoa in each tube and incubated for 20 s and 30 min with either BWW medium (serving as control) or SAF (150 *μ*g). Further, to evaluate receptor mediated blockage of SAF induced apoptosis, another set of experiment was carried out using different concentrations of receptor, namely, (c) SAF (150 *μ*g) + receptor (125 *μ*g) or (d) SAF (150 *μ*g) + receptor (150 *μ*g).

*Annexin V-PI Assay.* The anticoagulant Annexin V preferentially binds to negatively charged phospholipids such as phosphatidylserine [12]. By conjugating fluorescein to Annexin V, it has been possible to use the marker to identify apoptotic cells by flow cytometry. During apoptosis, the cells bind Annexin V prior to the loss of the plasma membrane's ability to exclude propidium iodide (PI). Therefore, by staining cells with a combination of Annexin V and PI, it is possible to simultaneously distinguish live, apoptotic, and necrotic sperm populations.

Staining with Annexin V-PI was performed using a commercial kit (Annexin V-FITC Apoptosis detection kit, Sigma Chemical). In brief, after the completion of incubation at 37°C, aliquots of semen samples containing 4 × 10^6^ sperm/mL were suspended in 500 *μ*L of binding buffer, labeled with 10 *μ*L of annexin V-FITC plus 5 *μ*L of PI, incubated for 10 min in the dark, according to the manufacturer's instructions, and immediately analysed by flow cytometry. Samples were evaluated by FL-1 (Annexin V-FITC) and FL-3 (PI) detectors. The cell population of interest was gated on the basis of the forward and side-scatter properties. The different labelling patterns in the bivariate Annexin-V PI analysis identified the different cell populations where FITC negative and PI negative were designated as viable cells; FITC positive and PI negative as apoptotic cells; and FITC positive and PI positive as late apoptotic or necrotic cells.

*Flow Cytometry. *Flow cytometry analysis was performed using the flow cytometer BD Canto II. Ten thousand events were measured for each sample at a flow rate of 50–100 events/s and analysed using the BD DIVA Software. The debris was gated out, by drawing a region on the forward versus side-scatter dot plot enclosing the population of cells of interest. The compensations and the settings were adapted according to the assay utilized.

#### 2.6.3. Fertility Outcome

Sexually mature female Balb/c mice were given intravaginal administration of different concentrations of SAF. Animals were divided in 5 groups, and each group received a single dose of 1, 2.5, 5, 10, 20, or 30 *μ*g/20 *μ*L during proestrus-estrus transition phase (*n* = 5 group). All the animals were allowed to mate overnight with proven fertile males in a ratio of 2 : 1. After confirmation of mating (presence of vaginal plug), female mice were separated and were observed for consistent weight gain as an indicator of pregnancy. Control group receiving PBS alone was preceded in the same way.

 SAF at a concentration of 5 *μ*g when administered intravaginally before mating led to infertility. To evaluate the receptor mediated blockage of SAF induced infertility, female Balb/c mice were divided into two groups. Group I received 5 *μ*g of SAF alone while Group II was divided into four subgroups, each receiving 5 *μ*g of SAF in combination with 5, 10, 20, or 30 *μ*g of receptor (*n* = 5/group). The control group received 20 *μ*L of PBS alone. The mice were kept for mating as described above and were allowed to complete their pregnancies and observed for consistent weight gain.

Also, histological examination was carried out to validate the pregnancy related changes. For this, another set of mice were divided in three groups (*n* = 3/group) receiving PBS (control), SAF (5 *μ*g) or SAF (5 *μ*g), and receptor (20 *μ*g) in combination. All the mice were allowed to mate as described earlier, 1 mouse from each group was sacrificed on days 0, 7 and 14, and reproductive organs (ovaries ad uterus) were collected aseptically, fixed with 10% buffered formalin, then embedded in paraffin according to standard histological methods, stained with hematoxylin and eosin, and observed at 40x. 

### 2.7. Statistical Analysis

Each experiment was carried out thrice with samples from different donors. The data was analyzed by one-way analysis of variance (ANNOVA) using GraphPad Prism software (Version 6.0). A *P* value less than 0.05 was considered statistically significant.

## 3. Results

### 3.1. Purification of SAF and Sperm Receptor

Purification of sperm ligand was carried out using salt extraction method and column chromatographic techniques. Molecular weight estimation of purified SAF showed the molecular weight to be ~71 kDa. The receptor of interest, able to inhibit the agglutination and death caused by SAF *in vitro*, could be eluted by 2 M salt solution. Further, the receptor was concentrated using PEG 6000 under cold conditions and applied to Sephadex G-200 column for purification. Molecular weight of receptor was found to be ~125 kDa.

#### 3.1.1. MALDI-TOF of SAF and Sperm Receptor

**Figure 1:**
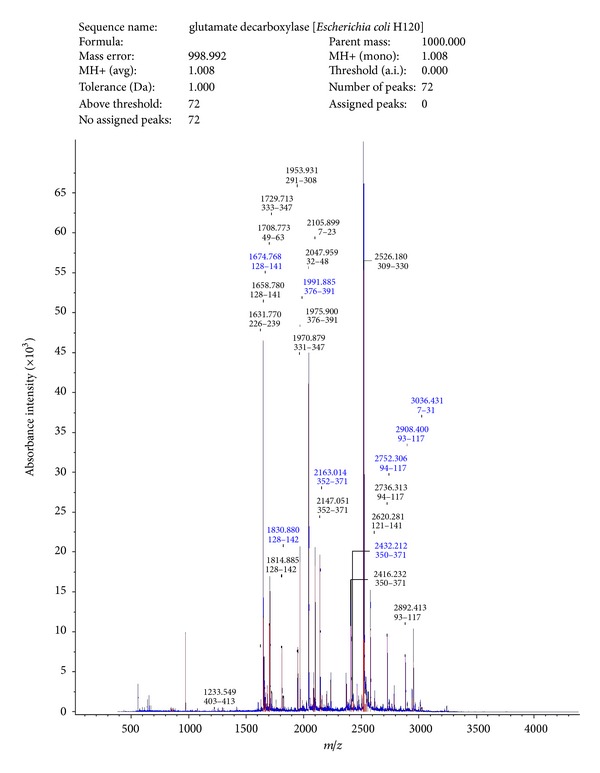
MALDI-TOF analysis of SAF.

**Figure 2:**
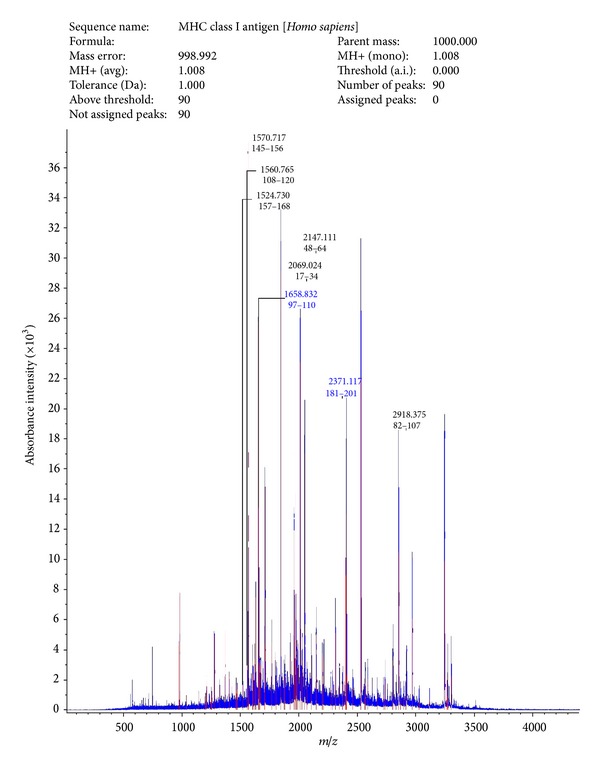
MALDI-TOF analysis of sperm receptor.

Peptide spectra analysis report showed that SAF had homology with glutamate decarboxylase from *Escherichia coli* H120 (Figure 1), and spectra analysis report of receptor indicated homology with MHC class I antigen of *Homo sapiens* (Figure 2).

### 3.2. Receptor Mediated Sperm Impairment by SAF

#### 3.2.1. Mg^2+^ Dependent Sperm ATPase Activity

The effect of SAF on the Mg^2+^ dependent ATPase activity of pooled and sonicated human spermatozoa was determined. From the results, it could be observed that SAF could significantly inhibit Mg^2+^ dependent ATPase activity of spermatozoa in a dose dependent manner. At a concentration of 80 *μ*g, there was decrease from 1580 ± 2.0 (control) ATPase units to 812.67 ± 2.52 (*P* < 0.01) ATPase units within 1 h of incubation with SAF (Table 1). In the presence of receptor, effect of SAF was inhibited as receptor (100 *μ*g) could raise the ATPase units from 812.67 ± 2.52 to 1472.67 ± 2.08 (*P* < 0.001) comparable to control (1580 ± 2.0) indicating that receptor is dependent on SAF (Table 2).

#### 3.2.2. Apoptosis

**Figure 3:**
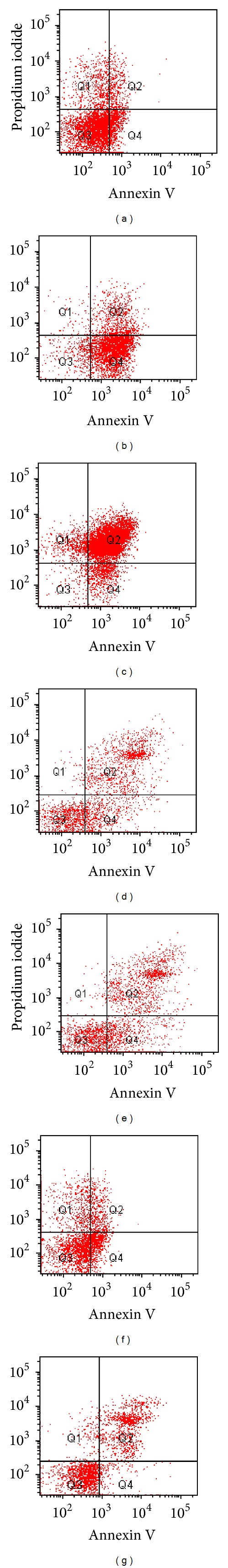
Representative cytogram for detection of apoptotic and necrotic sperm when exposed to the following for 0 or 30 min. (a) Spermatozoa alone, (b) SAF (150 *μ*g) for 0 min, (c) SAF (150 *μ*g) for 30 min, (d) SAF  +  receptor (125 *μ*g) for 0 min, (e) SAF + receptor (125 *μ*g) for 30 min, (f) SAF + receptor (150 *μ*g) for 0 min, (g) SAF + receptor (150 *μ*g) for 30 min. Sample cytogram stained using Annexin V/PI staining showing necrotic cells in Quadrant Q1, late apoptotic in Q2, live cells in Q3 and early apoptotic in Q4.

Incubation of SAF with spermatozoa increased significantly the percentage of apoptotic spermatozoa with phosphatidyl serine (PS) externalization compared to spermatozoa incubated without SAF. SAF at concentration of 150 *μ*g significantly decreased percentage of live cells from 75.8 ± 1.2% (control) to 28.1 ± 0.8% (*P* < 0.01) and shifted a higher percentage (58.3 ± 2.3%) towards early apoptotic stage (Figures 3(a) and 3(b)) in 0 min. Further incubation of spermatozoa with SAF for 30 min, led to a significant increase in percentage of late apoptotic and necrotic spermatozoa to 70.6 ± 0.9% leaving behind a live population of mere 5 ± 1.3% (*P* < 0.001) (Figure 3(c)). Simultaneous incubation of spermatozoa in the presence of SAF (150 *μ*g) and receptor (125 *μ*g) showed a noteworthy decrease in the percentage of apoptosis along with a concomitant increase in percentage of live spermatozoa both at 0 (58.1 ± 2.34%) and 30 min (55.8 ± 0.23%) (*P* < 0.01), respectively (Figures 3(d) and 3(e)). At a higher concentration of 150 *μ*g, receptor further added the protective effect towards spermatozoa by maintaining the percentage of viable cells at 65.2 ± 1.67% and 59.5 ± 1.56% at 0 and 30 min, respectively (Figures 3(f) and 3(g)).

#### 3.2.3. Fertility Outcome

Single administration of SAF at a concentration of 5 *μ*g or above led to blockage of conception. Groups receiving <5 *μ*g SAF remained fertile and delivered pups alike to control group with consistent weight gain. Different doses of receptor in combination with SAF were administered intravaginally to observe its effect on fertility outcome. Results showed that receptor when given at a concentration of ≥20 *μ*g led to conception alike to control group indicating the possible link of receptor-ligand interaction between receptor and SAF.

**Figure 4:**
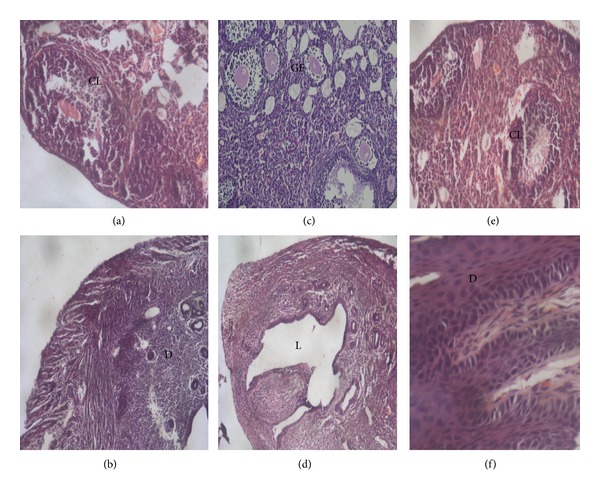
Histology of female reproductive organs (ovary and uterus) on day 14 in mice treated with PBS ((a), (b)), SAF ((c), (d)), and SAF + receptor ((e), (f)). Ovary showing the presence of corpus luteum (CL) in sections (a) and (e) while section (c), that is, SAF treated ovary at diestrus, showing only the presence of Graafian follicles (GF). Presence of decidua (D) was also observed in uterus.

Further, histological examination was carried out to observe pregnancy related changes on days 0, 7, and 14. Presence of corpus luteum was observed on day 14 in ovary of both control and combination group (SAF + receptor) in contrast to SAF treated groups (Figures 4(a) and 4(e)). Thickening of lining of uterus was present in addition to decidua formation in both groups (Figures 4(b) and 4(f)) except for the latter one indicating the absence of any pregnancy related changes in SAF treated mice (Figures 4(c) and 4(d)).

## 4. Discussion

Successful fertilization requires a sperm plasma membrane with normal integrity and function [13]. The numerous functions of the membrane are related to cell metabolism, for maintaining sperm motility, capacitation, acrosome reaction, and sperm-oocyte interaction. Acute and chronic infections and consequent inflammation in the male reproductive system may compromise the sperm cell function and the whole spermatogenetic process [1], causing qualitative and quantitative sperm alterations. Recent studies have shown that the simple presence of bacteria in semen samples may compromise the sperm quality.

The most frequently isolated microorganism in male patients with genital tract infections or semen contamination is *Escherichia coli*. The negative influence of this species on sperm quality is partially due to its effect on motility [5]. Ultrastructural damage of spermatozoa induced by *E. coli* has been discovered by use of different electron microscopy techniques. Rapidity and extent of sperm *E. coli* interaction indicate strong adhesive forces pointing towards receptor-ligand interaction [6]. Although a number of studies have evaluated the ability of *E. coli* to affect sperm motility by adherence and agglutination, very few have identified the molecular players playing role behind this interaction. 

In our laboratory, a sperm agglutinating factor (SAF) has been isolated and purified from *E. coli* which impedes sperm motility by agglutination and is spermicidal at higher concentrations [7]. Scanning electron microscopic studies have revealed that, apart from being spermicidal, the factor could also induce morphological defects on spermatozoa. Using SAF as a tool, the corresponding receptor was isolated and purified from human spermatozoa which could block SAF induced negative effects on spermatozoa [8]. Furthermore, an attempt was made to establish the identity of both SAF and receptor using MALDI-TOF. Peptide mass finger printing using MALDI-TOF revealed sequence similarity of purified SAF to glutamate decarboxylase and that of receptor to MHC class I. Interaction between the two molecules has already been reported in case of diabetes mellitus where MHC class I restricted determinants have been found on glutamic acid decarboxylase 65 molecule capable of inducing spontaneous CTL activity [14]. However, to the best of our knowledge, no correlation has been drawn between these molecules with respect to sperm bacteria interactions. Furthermore, in the present study, effect of SAF on different sperm parameters, namely, Mg^2+^ dependent ATPase and apoptosis, was studied and receptor-dependent interactions can be confirmed if they are competitively inhibited by addition of the specific receptor component. 

Spermatozoa are immotile in the testis and become motile upon their release into the external medium. Motility of spermatozoa depends on the dynein ATPases which hydrolyse ATP to produce flagellar beat [15]. During activation, the change in motility parameters seems to be directly related to ATP content. In this study, we observed a negative correlation between the SAF concentration and Mg^2+^ dependent ATPase activity which was measured as the inorganic Pi content released upon breakdown of ATP by ATPases. The activity decreased from 1580 ± 2.0 units in control to 812.67 ± 2.52 (*P* < 0.01) units upon incubation with SAF. 

Coincubation of spermatozoa with sperm receptor showed a remarkable increase in reduction of ATP which was dose dependent, as the ATPase units rose from 812.67 ± 2.52 to 1472.67 ± 2.08 (*P* < 0.001) with increasing concentrations of receptor. These results highlight the receptor-ligand interaction between SAF and corresponding sperm receptor.

Apoptosis occurs during normal spermatogenesis and plays a role in controlling sperm population. On the other hand, apoptosis may also be induced by several environmental, chemical, and physical factors including UV radiation, anticancer drugs and temperature changes [16]. The factors that could initiate apoptosis in spermatozoa are unknown; however some authors have observed that apoptosis can be stimulated by the direct contact with bacteria and its products even in the presence or absence of reactive oxygen species (ROS) [17]. Recently, it has been shown that experimentally induced *E. coli* infection causes sperm apoptosis independent of reactive oxygen species. Lipopolysaccharides and porins of Gram-negative bacteria have been shown to have a similar effect on sperm [18]. 

Apoptosis is associated with loss of membrane asymmetry leading to externalization of phosphatidyl serine (PS) on the outside of plasma membrane. Annexin V is a phospholipid-binding protein with high affinity for PS and identifies cells with deteriorated membranes, which is one of the earliest features of cells undergoing apoptosis. PI is a vital dye that is used to measure viability and to distinguish apoptotic from necrotic cells. Therefore, Annexin V is not an absolute marker of apoptosis [19], and, hence, it is often used in conjunction with PI, which binds to nucleic acids that can penetrate the plasma membrane when membrane integrity is disturbed.

*In vitro* incubation of SAF (150 *μ*g) and spermatozoa was carried out to investigate whether apoptosis was involved in loss of viability induced by SAF. Interestingly, upon incubation with SAF, significant differences were observed in the percentages of live and apoptotic spermatozoa even at 0 min (*P* < 0.001). SAF immediately shifted a majority of live sperm population to late apoptotic stage as significantly higher number of sperm had membrane PS externalization. After 30 min, an increased and elevated number of sperm shifted to late apoptotic stage as shown by PI staining indicating that great population of cells had undertaken the pathway towards late apoptosis. It is noteworthy that early and late apoptotic effects were observed with SAF (isolated from *E. coli*), and this ability of SAF to cause sperm apoptosis within short span of time suggests that sperms may also be damaged during their transit in an infected female genital tract. Also, the reported sperm apoptotic effects of incubation with SAF may be added to the direct detrimental effects on other sperm parameters. 

Further, this SAF induced apoptosis could be blocked by simultaneous addition of receptor, which at a concentration of 125 *μ*g reduced the apoptotic population to 22.2%, thereby preserving the live population at 58.1%. This effect did not diminish with time as, even after 30 min of incubation, the live population was maintained at much higher percentage. Also, at higher concentration of receptor, that is, 150 *μ*g, the percentage of live cells was almost comparable to control as expected. Therefore, we speculated that apoptosis may be involved in sperm death induced by SAF, and this interaction can be blocked by specific interactions existing between SAF and sperm receptor.

From the results obtained so far, we found that SAF has detrimental effects on the above-mentioned sperm parameters which play important role in successful fertilization. Further, SAF induced adverse effects could be nullified using receptor* in vitro*. So, an attempt was made whether similar line can be drawn between *in vitro *and *in vivo *studies, and it was observed that simultaneous administration of receptor at a concentration of ≥20 *μ*g with SAF (5 *μ*g) led to conception as observed in control groups receiving PBS alone. Histological examination also confirmed the presence of pregnancy related changes in control and receptor treated group in contrast to SAF treated group. These results further indicate the presence of receptor-dependent action of SAF both *in vitro *as well as *in vivo.*

## 5. Conclusion

Our findings establish that SAF can compromise various sperm parameters such as motility, viability, and Mg^2+^ dependent ATPase activity and could lead to infertility, which could be reversed by the addition of purified receptor indicating that sperm impairment and infertility induced by SAF are receptor mediated. 

## Supplementary Material

Quantitative analysis of apoptotic and necrotic spermatozoa after 30 min of incubation with either of SAF (150*μ*g) or SAF (150*μ*g) + receptor (150*μ*g). Data are mean ± SEM of three different experiments (p < 0.001).

## Figures and Tables

**Table 1:** Effect of SAF on Mg^2+ ^dependent ATPase activity.

S. No.	Concentration of SAF (*µ*g)	Mg^2+ ^ATPase activity (units)
Control	—	1580 ± 2.0
(1)	5	1560.33 ± 2.51
(2)	10	1405 ± 2.65
(3)	20	1265.67 ± 1.53
(4)	40	1040 ± 2.0
(5)	80	812.67 ± 2.52*

The results are expressed as mean ± SD (*P < 0.001).

**Table 2:** Effect of receptor on SAF induced Mg^2+^ dependent ATPase activity.

S. No.	Concentration of receptor (*µ*g)	Mg^2+ ^ATPase activity (units)*
Control	—	1580 ± 2.0
(1)	50	844.33 ± 2.08
(2)	75	1208.33 ± 1.53
(3)	100	1472.67 ± 2.08

*The results are expressed as mean ± SD.
