# Supplementary material for: Sperm Impairment by Sperm Agglutinating Factor Isolated from Escherichia coli: Receptor Specific Interactions
Source: Biomed Res Int. 2013 Jul 17;2013:548497. doi: 10.1155/2013/548497 (PMC3730385; doi:10.1155/2013/548497)
Supplement: Supplementary file 1 — Quantitative analysis of apoptotic and necrotic spermatozoa after 30 min of incubation with either of SAF (150μg) or SAF (150μg) + receptor (150μg). Data are mean ± SEM of three different experiments (p < 0.001). [file 548497.f1.doc]

**
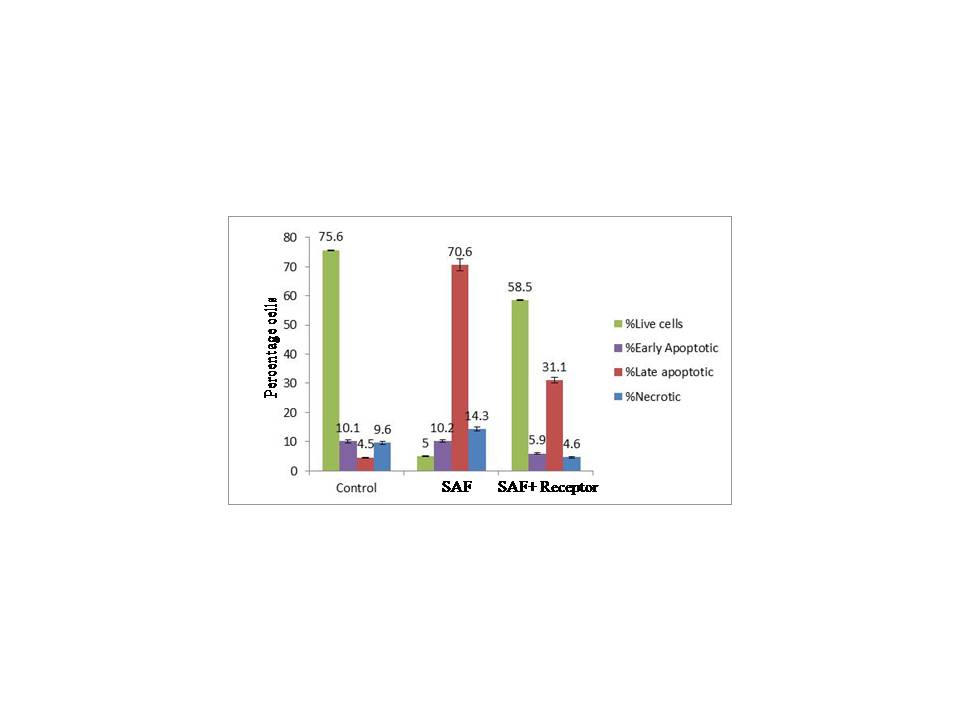
**

**Figure S1.** Quantitative analysis of apoptotic and necrotic spermatozoa after 30min of incubation with either of Control (BWW), SAF (100µg) or SAF (100µg) + receptor (125µg). Data are mean ± S.D. of three different experiments (*p* < 0.001).
